# Supplementary material for: Canyoning Accidents in Austria from 2005 to 2018
Source: Int J Environ Res Public Health. 2019 Dec 22;17(1):102. doi: 10.3390/ijerph17010102 (PMC6982325; doi:10.3390/ijerph17010102)
Supplement: Supplementary file 1 [file ijerph-17-00102-s001.zip › ijerph-649020-supplementary/Table S1 NACA Scoring.docx]

**Table S1:** NACA Scoring (National Advisory Committee for Aeronautics)

**NACA-Scoring (National Advisory Committee for Aeronautics) [19]**

NACA 0 No injury or illness

NACA 1 Injuries/diseases without any need for acute physician care

NACA 2 Injuries/diseases requiring examination/therapy by a physician, but hospital admission is not indicated

NACA 3 Injuries/diseases without acute threat to life, but requiring hospital admission

NACA 4 Injuries/diseases that can possibly lead to deterioration of vital signs

NACA 5 Injuries/diseases with acute threat to life

NACA 6 Injuries/diseases transported after successful resuscitation of vital signs

NACA 7 Lethal injuries or diseases (with or without resuscitation attempts)
